# Comparison of Visual Fixation Trajectories in Toddlers with Autism Spectrum Disorder and Typical Development: A Markov Chain Model

**DOI:** 10.3390/brainsci12010010

**Published:** 2021-12-23

**Authors:** Francesco Masedu, Roberto Vagnetti, Maria Chiara Pino, Marco Valenti, Monica Mazza

**Affiliations:** 1 Department of Applied Clinical Sciences and Biotechnology, University of L’Aquila, 67100 L’Aquila, Italy; francesco.masedu@univaq.it (F.M.); mariachiara.pino@univaq.it (M.C.P.); marco.valenti@univaq.it (M.V.); monica.mazza@univaq.it (M.M.); 2 Regional Reference Centre for Autism of the Abruzzo Region, Local Health Unit ASL 1, 67100 L’Aquila, Italy

**Keywords:** autism spectrum disorder, Markov model, eye-tracking, transition probabilities, dynamic visual search

## Abstract

Autism spectrum disorder (ASD) is a neurodevelopmental condition in which visual attention and visual search strategies are altered. Eye-tracking paradigms have been used to detect these changes. In our study, 18 toddlers with ASD and 18 toddlers with typical development (TD; age range 12–36 months) underwent an eye-tracking paradigm where a face was shown together with a series of objects. Eye gaze was coded according to three areas of interest (AOIs) indicating where the toddlers’ gaze was directed: ‘Face’, ‘Object’, and ‘No-stimulus fixation’. The fixation sequence for the ASD and TD groups was modelled with a Markov chain model, obtaining transition probabilities between AOIs. Our results indicate that the transition between AOIs could differentiate between toddlers with ASD or TD, highlighting different visual exploration patterns between the groups. The sequence of exploration is strictly conditioned based on previous fixations, among which ‘No-stimulus fixation’ has a critical role in differentiating the two groups. Furthermore, our analyses underline difficulties of individuals with ASD to engage in stimulus exploration. These results could improve clinical and interventional practice by considering this dimension among the evaluation process.

## 1. Introduction

Researchers have reported the extraordinary ability of infants to detect social stimuli [1,2]. In addition, some authors [3,4,5] have shown that infants can efficiently detect faces within complex visual scenes. Face perception is an important capacity that allows us to interact with other people and is a precursor of complex social competence such as emotion recognition, theory of mind, empathy, etc. [6,7]. Researchers have generally used the visual search paradigm to evaluate the capacity to detect and perceive a stimulus. This paradigm is based on the idea that if attention is drawn automatically to the target stimulus then the search time will be unaffected, or minimally affected, by the number of distractors that are present [8]. The power of the face to capture attention has been demonstrated extensively in infants and adults [9,10,11,12]. Among multiple objects shown representing different stimuli, children prefer faces, with no decrease due to habituation to the stimulus; moreover, they spend more time observing correctly oriented faces [13]. Researchers have shown that the preferences for faces seems already present in newborns [14,15]; it is also hypothesized that the preference is related to visual patterns consistent with a human looking at them [16]. In the worldwide general population, the autism spectrum disorder (ASD) prevalence is estimated to be around 1%, based on screening and register-based studies [17].

The literature indicates that children with ASD have an atypical visual perception profile early in their lives which influences their visual responsiveness, distribution of attention and social orienting. This is consistent with a study by Webb and collaborators [18]: they found that early signs of risk of subsequent ASD diagnosis include decreased use of facial information, including failure to look at the faces of social partners and failure to use eye gaze for joint attention. Individuals with ASD are characterized by altered and impaired processing of social information [19,20,21]. 

Di Giorgio et al. [1] found that a lower preference for social stimuli seems to be present in newborns with a high risk of an ASD diagnosis. Frank et al. [22] reported that during the first year of life of typical development, the proportion of time infants spend gazing at faces that are part of complex displays increases considerably and that visual attention is a key element to find stimuli of interest in a complex scenario. However, visual attention in individuals with ASD is perseverative (longer fixations per image explored), detail oriented and less explorative with regard to both social and non-social stimuli, suggesting that it could represent a generalized impairment [23]. Hosozawa and colleagues [24], using short video clips taken from TV programs or films for children, found that the looking patterns of children with ASD are heterogeneous; in addition, they looked less at the faces and easily looked away from protagonists. Chawarska et al. [25] added evidence that individuals with ASD pay increased attention to non-social objects. Several studies have highlighted that individuals with ASD are characterized by atypical patterns of social stimuli [26,27].

Another important feature of object preference seems to be based, as highlighted by Sasson and Touchstone [28], on the ways faces are matched with certain object categories (e.g., vehicles), so that children with ASD gazed upon faces less than the objects. Otherwise, individuals with ASD showed preferences comparable to children with typical development (TD) [28]. 

Individuals with ASD have less frequent saccades compared to individuals with TD and they prefer to pay attention to the mouth when they look at human faces [29]. Literature indicates that individuals with ASD show altered eye trajectories compared to individuals with TD [30]. Carette and colleagues [31] used a machine learning approach to show that, when individuals with ASD look at video recordings, their scan path could be identifiable with high accuracy. Jiang et al. [32] found that individuals with ASD have different response times and eye trajectories during an emotion recognition task. Differences in eye gaze behavior were used to distinguish the two groups through a random forest classification algorithm. Vu et al. [33] used a clustering algorithm to underline that gaze pattern distribution of children viewing a social scene, human face, or objects could distinguish between ASD and TD groups with high accuracy. In addition, their work suggests that differences can be detected through reduced exposure to the stimulus, i.e., they reached good accuracy with an exposure of 5 s.

In the literature, many models have been implemented to distinguish between individuals with ASD and TD based on eye-gaze metrics. For an in-depth discussion, we shall refer to a recent review that reports on machine learning methods used in various studies [34].

Fixations can be modelled in terms of a time sequence in which the events described are the fixations on objects. A description of these sequences can provide useful information about the behavior of individuals in terms of visual search patterns associated with certain disorders. The work of Treisman and Gelade [8] supported the idea to study a visual search sequence using a Markov chain. A Markov chain is a stochastic process that provides, given a set of states, the probability of moving from one state to another based on the events that occur [35] at the present time, regardless of the previous history. Thus, given the present state of the fixations measured by eye trackers, a Markov chain could describe the probability of displacing the gaze from one given starting stimulus to another. Markov chains could model the probability distribution of the gaze state according to transition matrix among areas of interest (AOIs) by using eye-tracking information. This approach could provide further insights into factors underlying scan paths during visual search. Markov models have been exploited in eye tracker data analysis. For example, Ulutas and collaborators [36] investigated fixation sequences of expert quality control operators compared with novice operators. Kim and colleagues [37] found that Markov models are well suited for paradigms that contain moving stimuli. Jansen et al. [38] reported which reading strategies are used by experts who read algebraic expressions. Moreover, Markov models have been used to predict human eye fixations [39] and to highlight differences in scan path patterns among subjects [40]. Alie et al. [41] applied Markov models on eye gaze patterns of 6-month-old infants at risk of autism, considering when they looked at their mothers’ faces and when they looked away. After a training session, they found that the model could correctly classify children who received a future ASD diagnosis and a control group with an accuracy of about 93%. An important aspect of this is that differences in eye gaze patterns are already identifiable at early age.

We decided to characterize the dynamic structure of the eye-tracking process (i.e., the transition matrix) and to compare groups in terms of the transition pattern between the states of the process. This choice privileges the comparison of the dynamic behavior of groups, instead of the comparison of simple parameters describing overall properties or single time points related. This feature is more consistent with an exact characterization of the process instead of its specific properties that turn out to be a consequence of the transition pattern. 

Our study focuses on the Markov model, the strength of this approach is that it considers that what an individual is currently observing influences what it is likely she/he is going to observe. Furthermore, another strength is that the model obtained from the analysis is analyzed to describe behavioral differences between individuals with ASD and TD.

The aim of our study was to compare the eye gaze transitions of children with ASD or TD by using Markov chains in an eye-tracking setting. We tested the hypothesis that Markov chains are a suitable strategy to model eye-tracking paradigms, allowing discrimination between children with ASD or TD based on eye gazes, which accounts for the visual search pattern.

## 2. Method

### 2.1. Participants 

Demographic and clinical information on all participants is summarized in Table 1. Thirty-six toddlers participated in the study: 18 with ASD (age range 12–36 months) recruited at the Reference Centre for Autism of the Abruzzo Region of Italy [17] and 18 with TD (age range 12–36 months) recruited from a local nursery in the same region and matched to the ASD group with respect to chronological age. ASD diagnoses were made by experienced clinicians according to the criteria of the *Diagnostic and Statistical Manual of Mental Disorders* (5th ed. [42]). ASD diagnoses were confirmed by using the Autism Diagnostic Observation Schedule, Second Edition (ADOS-2 [43]). We used the ADOS-2 Toddler Module for children under 30 months of age (eight children) and the ADOS-2 Module 1 for older children (10 children). Each of the eight children were classified as at risk according to the Toddler Module, and since the data acquisition for this study, they have been confirmed as ASD cases during the follow-up clinical evaluations. The gender distribution between groups was homogeneous (ASD: 14 males and 4 females; TD: 11 males and 7 females) according to Fisher’s exact test (*p* = 0.47).

Ethical approval was obtained from the Ethics Committee of the Local Health Agency. The Ethics Committee approved the experimental protocol under number 186061/17. We obtained informed consent from the holders of parental rights. 

### 2.2. Experimental Paradigm

**Figure 1:**
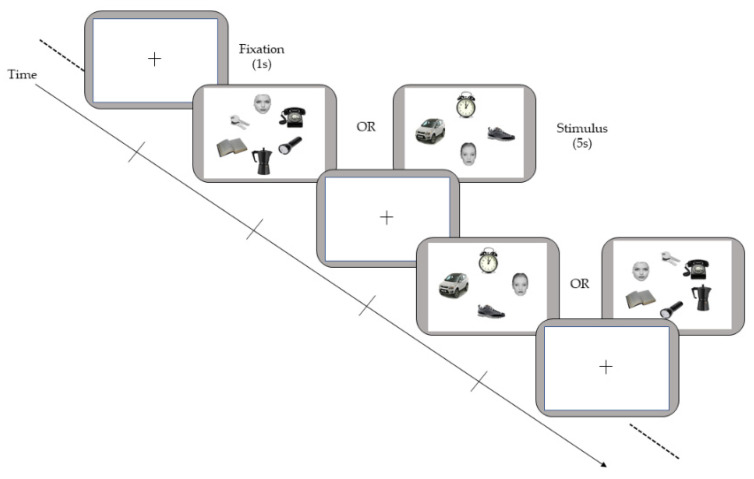
Illustration of the paradigm employed in the experiment.

The task was a visual exploration paradigm constructed by referring to the study of Sasson et al. [23], presenting social stimuli among many non-social stimuli to children with ASD and TD. We decided to expose children to a reduced number of stimuli to be more coherent with a potential clinical or intervention situation. The task stimuli comprised two sets of 16 displays, 16 simple configurations consisting of four items each (a target face and three different objects, e.g., a car, a shoe and an alarm clock) and 16 complex configurations consisting of six items each (a target face and five different distractor objects, e.g., a car, a shoe, an alarm clock, a book and keys), for a total of 32 slides. We administrated images with a different ratio of stimuli type (4-item set and 6-item set) to reduce expectancy effects and to expose participants to a varying degree of distractors. We counterbalanced these two types of images. The presentation of the stimuli had been randomized to adjust for order bias. The face location was changed within each set of images (in the 4-item set and the 6-item set) to prevent distortion of results due to positioning. Thus, we presented the face an equal number of times in every possible position in images with four items, and at least two times in images with six items. In the latter case, we also randomly chose the third presentation in a position before the assessment of each participant. Displays were presented for 5 s; between the displays, a fixation point was presented in the center of the screen, ensuring that the gaze was directed to the center until the next display was presented. Based on the literature, Fletcher-Watson et al. [30] found differences between ASD and TD eye-gaze path by exposing them to 3 s of stimuli. Furthermore, the study of Vu et al. [33] suggests that an exposition of 5 s optimizes the discrimination between groups. We used a 5 s exposition time for each stimulus to avoid children’s loss of attention or interest in the task, as they underwent a passive paradigm. The stimuli were comparable in size and were arranged on a circular grid, on a white background, at an equal distance from the center of the screen. A chart of the experiment is reported in Figure 1.

### 2.3. Apparatus 

The task was performed by using the Tobii T120- Eye Tracker equipment (Danderyd, Sweden) consisting of a GL-2760-LED backlight monitor (Eindhoven, Netherlands) with a resolution of 1920 × 1080 pixels, which both presented the stimuli and recorded the gaze. This eye-tracking system is non-invasive and the subject has little indication that eye movements are being tracked; artificially constraining head movements is not required. The system tracks both eyes to an accuracy of 0.5 degrees at a sampling rate of 60 Hz. The Tobii equipment was connected to a laptop computer that was used to run the tasks. Calibration procedures, stimulus creation, data acquisition and visualization were performed by using the Tobii Studio™ Analysis Software (version 3.4.5; Danderyd, Sweden). 

### 2.4. Procedure 

All toddlers were tested once in a quiet, darkened room. Children sat on their caregivers’ lap. We asked the caregivers to cover their eyes with a blindfold to avoid gauging their gaze. The experiment started with a calibration phase that was immediately followed by the test phase. During calibration, a cartoon was presented in the center of the screen. When the infant started to look at the smiley face, it moved to the top left corner of the screen and remained in this position until the toddler fixated on it. Then, the smiley face moved to the bottom right corner and remained in that position. These three positions were used to compute the pupil–corneal reflection from three points on the screen, allowing the system to derive the gaze direction during the test phases. The calibration accuracy was checked and the calibration procedure was repeated if necessary. 

**Figure 2:**
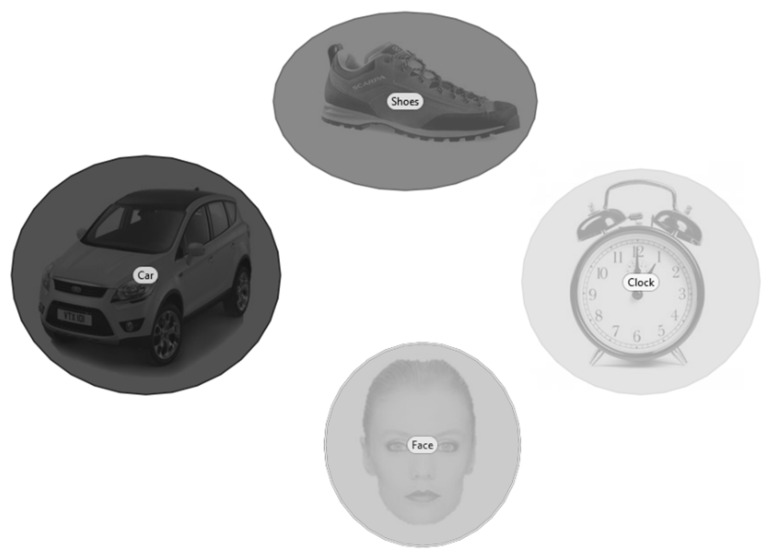
Example of AOIs.

After the calibration phase, the participant was presented with 32 randomly selected displays. Ellipsoid AOIs were defined manually for each image in the displays. AOIs were defined to fully cover items in the image. We preferred larger AOIs rather than tightened AOIs as they mitigate differences between different methods [44]. An AOI example is reported in Figure 2. At the end of the testing session, the participant received a reward (colored stickers). 

The fixation sequence on AOIs was then analyzed for each participant. The sequence of AOIs observed by participants was used to build the model. Thus, we extrapolated from eye-tracking data Fixation Index, which indicates the order in which a fixation event is recorded, and AOI activity for each AOI, which indicates if during the fixation event the fixation point was located inside that AOI or not. A fixation event was defined by the Tobii fixation filter (I-IV-filter) as any occasion when the direction of gaze remained within 0.5 degrees of the visual angle for at least 100 milliseconds. Fixation events were coded as ‘Face’ when the fixation had the face stimulus as the target, ‘Object’ when the fixation had one of the objects presented in the slide as the target and ‘No-stimulus fixation’ when the gaze of the participant did not fall inside any AOI, but was still on the monitor.

## 3. Data Analysis 

Baseline statistics of the ADOS-2 Toddler Module and Module 1 have been provided in Table 1, comparing the ASD and TD groups for age and gender homogeneity using the *t*-test and Fisher’s exact test, setting the type I error at 5%. We built up a Markov chain characterizing the three AOIs described above as the possible states assumed in the chain. Indeed, the system status set has been defined: {face-gaze, object-gaze,no-stimulus-gaze}. The transition matrix for the ASD and TD groups have been estimated by maximising the loglikelihood function. Thus, we estimated the transition probabilities between states—AOIs—by using the sample relative frequencies with their corresponding standard error: ${\hat{p}}_{ij}^{MLE}=\frac{n_{ij}}{\sum_{u=1}^{k}n_{iu}}$ and ${\hat{se}}_{ij}=\frac{{\hat{p}}_{ij}^{MLE}}{\sqrt{n_{ij}}}$, where i, j = 1, 2, 3 [45]. 

In our setting, the transition matrices that define the chains associated with ASD and TD are designed such that the rows represent starting AOIs and the columns represent subsequent target AOIs. Thus, each matrix cell displays the transition probability to move from row AOI to column AOI. Graphs for the transitions have been provided.

We compared the ASD and TD groups by using two strategies. First, we addressed the gaze process difference between the ASD and TD groups by performing a divergence test for empirically estimated transition matrices, according to Kullback and Kupperman [46]. Second, we tested the difference between the steady states reached by the groups. The steady state probability vector represents a converging point of the distribution where the transition probabilities will no longer change; thus, it represents a sort of prediction, or attraction pattern, for the distribution of the states used to model the process. Differences emerging from the steady states could indicate a recurrent and constant difference between groups.

We carried out Markov property and Markov chain transition matrix analysis by using the statistical software R version 3.6.3 [47] and the statistical package *markovchain* [48]. Preliminarily, we tested for the Markov property of the two groups to check the feasibility of the devised model. We confirmed the Markov property for both groups: ASD (*χ*^2^ (27) = 36.2, *p* = 0.11) and TD (*χ*^2^ (27) = 35.9, *p* = 0.12). Thus, the hypothesis was verified. 

## 4. Results

**Figure 3:**
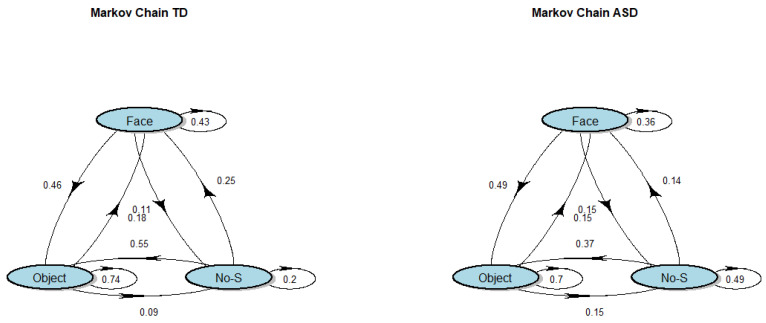
Graphical representation of Markov chains models of TD and ASD. Arrows represent transition direction. “No-S” stands for “No-stimulus fixation” state.

Transition matrices for each group are reported in Table 2. The cells indicate the probability to pass from row AOI to column AOI. Moreover, 95% confidence intervals are presented in parentheses. A graphical representation of the transition matrices is reported in Figure 3. The Markov chains associated with the ASD and TD groups are significantly different (*χ*^2^ (8) = 28.1, *p* < 0.001) confirming the hypothesis of different visual search behaviors.

Considering the lower and upper endpoints, we found that the TD group has a greater probability to be interested in faces (25%) or objects (55%) compared with the ASD group (14% and 37%, respectively). Moreover, the ASD group is more likely to continue to ‘not look’ at any of the proposed stimuli (49%) compared with the TD group (20%). We also found that the ASD group has a higher probability to pass from an object to not viewing a proposed stimulus (15%) compared with the TD group (8%).

**Figure 4:**
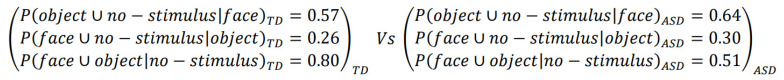
Comparison of departure probabilities between TD and ASD from one AOI to two other possible AOIs. Note. TD = Toddlers with Typical Development; ASD = Toddlers with autism spectrum disorder.

The analysis of transition matrices revealed a notable process difference between the groups. Looking at the probability to change the original state, as described by the transition matrices (Figure 4), we observed that for the states ‘face’ and ‘object’, the probability of departure from them is higher for the ASD group. The behavior was the opposite given the departure from the ‘no-stimulus fixation’ state, where the TD group showed a higher propensity to move away.

The steady state distributions show important differences between the groups. The TD group is more likely to look at faces (25%) or objects (65%) compared with the ASD group (19% and 59%, respectively). The ASD group has a higher probability to reach the ‘no-stimulus state’ (22%) compared with the TD group (10%) (Table 3). Moreover, there are two different probabilities of the hierarchy of states with respect to the ASD and TD groups, namely: $\langle object,\;face,\;no-stimulus\rangle_{TD}$ and $\langle object,\;no-stimulus,\;face\rangle_{ASD}$. These patterns show an inversion of the hierarchy of face versus no-stimulus in the ASD group.

## 5. Discussion

Research has generally used a visual research paradigm to appraise the capacity to detect and perceive stimuli. Given that a stimulus is strong enough to catch one’s attention, it turns out that distractors do not significantly affect the search time [8]. Regarding ASD, visual perception seems to be altered from early in life, characterized by a decreased interest in gazing upon faces and eyes [1,18], perseverative visual attention and a lack of visual exploration [23]. Our study partially agrees with this consolidated position. The statistical comparison of the transition matrices of the ASD and TD groups showed that they present different visual search strategies because the transition matrices describe different Markov chains [46]. Nonetheless, it is important to assess the statement of an overall difference and how this process works in discriminating the groups.

The transition matrices of the groups (Table 2) show that a large amount of variation between transition probabilities are accounted for if the starting state is ‘no-stimulus fixation’. In fact, from this state, the ASD group has a lower probability to see subsequent social or non-social stimuli compared with the TD group. It is important to emphasize that the ASD probability to remain in this particular state—that is, not focus attention on a social or non-social stimuli—is almost 50%, while for the TD group, it is 20%. Accordingly, in Figure 4, if a child with TD does not look at one of the proposed stimuli, they are more likely to look at one of them later (80%), while a child with ASD is less likely to look at them later (51%). These findings indicate that a child with TD will tend to change their eye gaze from this particular state to find other kinds of stimuli, contrary to ASD behavior.

Another issue worth mentioning is how the probability distribution of the steady state describes the capacity of the states to catch the gaze of children with ASD or TD. This capacity differentiates the groups in terms of the attractive strength of the state. The observed ordering behavior is consistent with the literature (Table 3), showing the low attitude of children with ASD towards faces, providing a discriminant property of the visual search process between children with TD or ASD. 

The two main results obtained seem consistent with each other; in addition, they provide a description of the different visual search patterns of the two groups. Our results indicate that from early ages, individuals with ASD are more likely to not be interested in the stimuli provided, given the occurrence of the ‘no-stimulus fixation’ state. This process pattern is of particular interest despite the well-known ASD attention difficulties [49,50]. As far as we know, the studies conducted so far have not considered this particular aspect, namely, when individuals with ASD do not focus on determined stimuli and how this affects the visual search process. Our study extends the existing literature because it describes and quantifies in probabilistic terms where gaze will fall according to the previous gaze. Furthermore, we have seen that these probabilities are different between the groups, leading to different gaze behavior. 

The analysis supports the hypothesis that the lack of attention to the proposed stimuli is a distinctive visual search behavior of children with ASD. Difficulties of visual attention are well known among the ASD population [51,52,53]. Our results are in line with the reduced flexibility in the control of visual attention as an early feature of autism, and the difficulties of individuals with ASD to switch the locus of attention [54]. Difficulties in attention disengagement have been reported [54], and our results are consistent with this hypothesis, underlining impairments in stimulus engagement, with reduced exploration of the environment from an early age. In addition, there are plausible cascade effects on the subsequent development. As a future perspective, it would be interesting to use this methodology on an older population to examine whether they could develop possible compensatory mechanisms [51] and whether the brain region connectivity involved in attention changes between childhood and adulthood in individuals with ASD [55].

There are a few limitations of this study. First, it would be appropriate to assess these results with a larger sample. Second, we categorized the stimulus spectrum according to three states. This approach, applied because of the small sample size, could be changed to encompass a wider set of possible states and could describe the visual search process in greater detail. 

## 6. Conclusions

This study is essentially explorative, suggesting a particular analytical setting. The overall analysis emphasizes the critical role played by the ‘no-stimulus state’, which describes the lack of directionality of the gaze. This state plays a major role in determining different visual processing patterns between individuals with ASD and TD, prompting the former group to explore the environment differently than the latter group. In addition, visual transition patterns could be considered during the diagnostic process, given the divergence in the visual search pattern of individuals with ASD from typical development. Moreover, visual transition patterns could provide meaningful insights into the efficacy of the clinical assessment of behavioral interventions. 

## Figures and Tables

**Table 1:** Demographic information for both groups and clinical data for the ASD group.

	TD (N = 18) Mean (SD)	ASD (N = 18) Mean (SD)	T	*p*
Age in month	29.7 (4.84)	30.7 (3.04)	−0.74	0.46
Gender	7 F; 11 M	4 F; 14 M		0.47
*ADOS-2 Toddler Module*				
Social Affect		15.2 (3.27)		
Restricted and Repetitive Behavior		3.40 (2.07)		
Total		18.6 (4.5)		
*ADOS-2 Module 1*				
Social Affect		12.7 (4.65)		
Restricted and Repetitive Behavior		4.23 (1.58)		
Total		17.0 (5.83)		

Note. TD = Toddlers with Typical Development; ASD = Toddlers with autism spectrum disorder; T = test statistic resulted from two independent samples *t* test; *p* = *p* values resulted from statistical test.

**Table 2:** Transition matrix of ASD and TD, lower and upper endpoints are reported between brackets (CI:95%).

	Group	FACE	OBJECT	NO-STIMULUS
FACE	TD	0.43 [0.40–0.46]	0.46 [0.43–0.50]	0.11 [0.09–0.12]
	ASD	0.36 [0.29–0.43]	0.49 [0.41–0.57]	0.15 [0.10–0.19]
OBJECT	TD	0.18 [0.16–0.19]	0.74 [0.71–0.76]	0.08 * [0.08–0.10]
	ASD	0.15 [0.13–0.18]	0.70 [0.65–0.76]	0.15 * [0.12–0.17]
NO-STIMULUS	TD	0.25 * [0.21–0.29]	0.55 * [0.49–0.60]	0.20 * [0.17–0.24]
	ASD	0.14 * [0.10–0.18]	0.37 * [0.30–0.43]	0.49 * [0.41–0.57]

Note. TD = Toddlers with Typical Development; ASD = Toddlers with autism spectrum disorder. * Denotes a difference between groups on the chance to pass from a row AOI to a column AOI.

**Table 3:** Steady state of TD and ASD (CI:95% are reported between brackets).

	FACE	OBJECT	NO-STIMULUS
TD	0.25 [0.24–0.26]	0.65 [0.64–0.66]	0.10 [0.09–0.11]
ASD	0.19 [0.17–0.21]	0.59 [0.56–0.62]	0.22 [0.20–0.24]

Note. TD = Toddlers with Typical Development; ASD = Toddlers with autism spectrum disorder.

## Data Availability

The datasets generated during and/or analyzed during the current study are available from the corresponding author on reasonable request.

## References

[1] Di Giorgio E., Frasnelli E., Salva O.R., Scattoni M.L., Puopolo M., Tosoni D. (2016). Corrigendum: Difference in Visual Social Predispositions Between Newborns at Low-and High-risk for Autism. Sci. Rep..

[2] Happé F., Frith U. (2014). Annual research review: Towards a developmental neuroscience of atypical social cognition. J. Child Psychol. Psychiatry.

[3] de Haan M., Johnson M.H., Halit H. (2003). Development of face-sensitive event-related potentials during infancy: A review. Int. J. Psychophysiol..

[4] Simion F., Di Giorgio E., Leo I., Bardi L. (2011). The processing of social stimuli in early infancy: From faces to biological motion perception. Prog. Brain Res..

[5] Valenza E., Leo I., Gava L., Simion F. (2006). Perceptual completion in newborn human infants. Child Dev..

[6] Mazza M., Mariano M., Peretti S., Masedu F., Pino M.C., Valenti M. (2017). The Role of Theory of Mind on Social Information Processing in Children with autism spectrum disorders: A Mediation Analysis. J. Autism Dev. Disord..

[7] Pino M.C., Mazza M., Mariano M., Peretti S., Dimitriou D., Masedu F., Valenti M., Franco F. (2017). Simple Mindreading Abilities Predict Complex Theory of Mind: Developmental Delay in autism spectrum disorders. J. Autism Dev. Disord..

[8] Treisman A.M., Gelade G. (1980). A feature-integration theory of attention. Cogn. Psychol..

[9] Bindemann M., Burton A.M., Hooge I.T., Jenkins R., de Haan E.H. (2005). Faces retain attention. Psychon. Bull. Rev..

[10] Di Giorgio E., Leo I., Pascalis O., Simion F. (2012). Is the face-perception system human-specific at birth?. Dev. Psychol..

[11] Fletcher-Watson S., Findlay J.M., Leekam S.R., Benson V. (2008). Rapid detection of person information in a naturalistic scene. Perception.

[12] Lewis M.B., Edmonds A.J. (2005). Searching for faces in scrambled scenes. Vis. Cogn..

[13] Gliga T., Elsabbagh M., Andravizou A., Johnson M. (2009). Faces attract infants’ attention in complex displays. Infancy.

[14] Johnson M.H., Dziurawiec S., Ellis H., Morton J. (1991). Newborns’ preferential tracking of face-like stimuli and its subsequent decline. Cognition.

[15] Valenza E., Simion F., Cassia V.M., Umiltà C. (1996). Face preference at birth. J. Exp. Psychol. Hum. Percept. Perform..

[16] Farroni T., Johnson M.H., Menon E., Zulian L., Faraguna D., Csibra G. (2005). Newborns’ preference for face-relevant stimuli: Effects of contrast polarity. Proc. Natl. Acad. Sci. USA.

[17] Valenti M., Vagnetti R., Masedu F., Pino M.C., Rossi A., Scattoni M.L., Mazza M., Eagle Group (2019). Register-based cumulative prevalence of autism spectrum disorders during childhood and adolescence in Central Italy. Epidemiol. Biostat. Public Health.

[18] Webb S.J., Neuhaus E., Faja S. (2017). Face perception and learning in autism spectrum disorders. Q. J. Exp. Psychol..

[19] Pino M.C., Masedu F., Vagnetti R., Attanasio M., Di Giovanni C., Valenti M., Mazza M. (2020). Validity of Social Cognition Measures in the Clinical Services for Autism Spectrum Disorder. Front. Psychol..

[20] Pino M.C., Vagnetti R., Masedu F., Attanasio M., Tiberti S., Valenti M., Mazza M. (2020). Mapping the Network of Social Cognition Domains in Children With Autism Spectrum Disorder Through Graph Analysis. Front. Psychiatry.

[21] Vagnetti R., Pino M.C., Masedu F., Peretti S., Le Donne I., Rossi R., Valenti M., Mazza M. (2020). Exploring the social cognition network in young adults with autism spectrum disorder using graph analysis. Brain Behav..

[22] Frank M.C., Amso D., Johnson S.P. (2014). Visual search and attention to faces during early infancy. J. Exp. Child Psychol..

[23] Sasson N.J., Turner-Brown L.M., Holtzclaw T.N., Lam K.S., Bodfish J.W. (2008). Children with autism demonstrate circumscribed attention during passive viewing of complex social and nonsocial picture arrays. Autism Res..

[24] Hosozawa M., Tanaka K., Shimizu T., Nakano T., Kitazawa S. (2012). How children with specific language impairment view social situations: An eye tracking study. Pediatrics.

[25] Chawarska K., Macari S., Shic F. (2012). Context modulates attention to social scenes in toddlers with autism. J. Child Psychol. Psychiatry.

[26] Pelphrey K., Sasson N.J., Reznick J.S., Paul G., Goldman B.D., Piven J. (2002). Visual scanning of faces in autism. J. Autism Dev. Disord..

[27] Senju A., Tojo Y., Dairoku H., Hasegawa T. (2004). Reflexive orienting in response to eye gaze and an arrow in children with and without autism. J. Child Psychol. Psychiatry.

[28] Sasson N.J., Touchstone E.W. (2014). Visual attention to competing social and object images by preschool children with autism spectrum disorder. J. Autism Dev. Disord..

[29] Sadria M., Karimi S., Layton A.T. (2019). Network centrality analysis of eye-gaze data in autism spectrum disorder. Comput. Biol. Med..

[30] Fletcher-Watson S., Leekam S.R., Benson V., Frank M.C., Findlay J.M. (2009). Eye-movements reveal attention to social information in autism spectrum disorder. Neuropsychologia.

[31] Carette R., Elbattah M., Cilia F., Dequen G., Guérin J.L., Bosche J. Learning to Predict Autism Spectrum Disorder based on the Visual Patterns of Eye-tracking Scanpaths. Proceedings of the 12th International Joint Conference on Biomedical Engineering Systems and Technologies—HEALTHINF.

[32] Jiang M., Francis S.M., Srishyla D., Conelea C., Zhao Q., Jacob S. Classifying individuals with ASD through facial emotion recognition and eye-tracking. Proceedings of the 2019 41th Annual International Conference of the IEEE Engineering in Medicine and Biology Society (EMBC).

[33] Vu T., Tran H., Cho K.W., Song C., Lin F., Chen C.W., Hartley-McAndrew M., Doody K.R., Xu W. Effective and efficient visual stimuli design for quantitative autism screening: An exploratory study. Proceedings of the 2017 IEEE EMBS International Conference on Biomedical & Health Informatics (BHI).

[34] Kollias K.F., Syriopoulou-Delli C.K., Sarigiannidis P., Fragulis G.F. (2021). The Contribution of Machine Learning and Eye-Tracking Technology in Autism Spectrum Disorder Research: A Systematic Review. Electronics.

[35] Gagniuc P.A. (2017). Markov Chains: From Theory to Implementation and Experimentation.

[36] Ulutas B.H., Özkan N.F., Michalski R. (2019). Application of hidden Markov models to eye tracking data analysis of visual quality inspection operations. Cent. Eur. J. Oper. Res..

[37] Kim J., Singh S., Thiessen E.D., Fisher A.V. (2020). A hidden Markov model for analyzing eye-tracking of moving objects. Behav. Res. Methods.

[38] Jansen A.R., Marriott K., Yelland G.W. (2007). Parsing of algebraic expressions by experienced users of mathematics. Eur. J. Cogn. Psychol..

[39] Zhong M., Zhao X., Zou X.C., Wang J.Z., Wang W. (2014). Markov chain based computational visual attention model that learns from eye tracking data. Pattern Recognit. Lett..

[40] Devlin S.P., Riggs S.L. (2017). Analyzing eye tracking data using a Markovian framework to assess differences in scan patterns. Proceedings of the Human Factors and Ergonomics Society Annual Meeting.

[41] Alie D., Mahoor M.H., Mattson W.I., Anderson D.R., Messinger D.S. Analysis of eye gaze pattern of infants at risk of autism spectrum disorder using markov models. Proceedings of the 2011 IEEE Workshop on Applications of Computer Vision (WACV).

[42] American Psychiatric Association (2013). Diagnostic and Statistical Manual of Mental Disorders.

[43] Lord C., Rutter M., DiLavore P.C., Risi S., Gotham K., Bishop S.L. (2012). Autism Diagnostic Observation Schedule.

[44] Hessels R.S., Kemner C., van den Boomen C., Hooge I.T. (2016). The area-of-interest problem in eyetracking research: A noise-robust solution for face and sparse stimuli. Behav. Res. Methods.

[45] Sison C.P., Glaz J. (1995). Simultaneous confidence intervals and sample size determination for multinomial proportions. J. Am. Stat. Assoc..

[46] Kullback S., Kupperman M., Ku H. (1962). Tests for Contingency Tables and Marltov Chains. Technometrics.

[47] R Core Team (2020). R: A Language and Environment for Statistical Computing.

[48] Spedicato G.A. (2017). Discrete time Markov chains with R. R J..

[49] Allen G., Courchesne E. (2001). Attention function and dysfunction in autism. Front. Biosci..

[50] Elsabbagh M., Holmboe K., Gliga T., Mercure E., Hudry K., Charman T., Baron-Cohen S., Bolton P., Johnson M.H., BASIS Team (2011). Social and attention factors during infancy and the later emergence of autism characteristics. Progress in Brain Research.

[51] Luna B., Doll S.K., Hegedus S.J., Minshew N.J., Sweeney J.A. (2007). Maturation of executive function in autism. Biol. Psychiatry.

[52] Townsend J., Harris N.S., Courchesne E. (1996). Visual attention abnormalities in autism: Delayed orienting to location. J. Int. Neuropsychol. Soc..

[53] van der Geest J.N., Kemner C., Camfferman G., Verbaten M.N., van Engeland H. (2001). Eye movements, visual attention, and autism: A saccadic reaction time study using the gap and overlap paradigm. Biol. Psychiatry.

[54] Elsabbagh M., Fernandes J., Webb S.J., Dawson G., Charman T., Johnson M.H., British Autism Study of Infant Siblings Team (2013). Disengagement of visual attention in infancy is associated with emerging autism in toddlerhood. Biol. Psychiatry.

[55] Farrant K., Uddin L.Q. (2016). Atypical developmental of dorsal and ventral attention networks in autism. Dev. Sci..

